# Association Between Serum 25-Hydroxyvitamin D and Blood Pressure in Young Adults

**DOI:** 10.3390/nu18050876

**Published:** 2026-03-09

**Authors:** Ching-Way Chen, Shu-Yu Tang, Yin-Yi Han, Sandy Huey-Jen Hsu, Jing-Shiang Hwang, Ta-Chen Su

**Affiliations:** 1 Graduate Institute of Clinical Medicine, National Taiwan University College of Medicine, Taipei 100229, Taiwan; brendon32402@gmail.com; 2 Division of Cardiology, Department of Internal Medicine, National Taiwan University Hospital Yunlin Branch, Yunlin 640203, Taiwan; boryangcafe@gmail.com; 3 Department of Anesthesiology, National Taiwan University Hospital, Taipei 100225, Taiwan; noviahan@gmail.com; 4 Department of Traumatology, National Taiwan University Hospital, Taipei 100225, Taiwan; 5 Department of Laboratory Medicine, College of Medicine, National Taiwan University Hospital, National Taiwan University, Taipei 100225, Taiwan; sandyhsu@ntuh.gov.tw; 6 Institute of Statistical Science, Academia Sinica, Taipei 115201, Taiwan; jshwang@stat.sinica.edu.tw; 7 Division of Cardiology, Department of Internal Medicine, Tungs’ Taichung MetroHarbor Hospital, Taichung 435403, Taiwan; 8 Division of Cardiology, Department of Internal Medicine, National Taiwan University Hospital, Taipei 100225, Taiwan; 9 Institute of Environmental and Occupational Health Sciences, College of Public Health, National Taiwan University, Taipei 100025, Taiwan; 10 Department of Environmental and Occupational Medicine, National Taiwan University Hospital, Taipei 100225, Taiwan

**Keywords:** 25-hydroxyvitamin D, blood pressure, young adults

## Abstract

Background: Vitamin D has been associated with blood pressure across the life course in observational studies, although effect sizes are generally modest and findings are not fully consistent. We examined the association between serum 25-hydroxyvitamin D [25(OH)D] concentrations and multiple blood pressure indices in a community-based cohort of young adults. Methods: We conducted a cross-sectional analysis within the Young Taiwanese Adults (YOTA) cohort, restricting the sample to adults aged 18–45 years with complete serum 25(OH)D and blood pressure data. Serum 25(OH)D was modeled as a continuous variable and additionally examined using predefined concentration categories. Systolic blood pressure (SBP), diastolic blood pressure (DBP), and mean arterial pressure (MAP) were assessed using standardized protocols. Multivariable linear regression models evaluated associations, with sequential adjustment for demographic, anthropometric, cardiometabolic, and lifestyle covariates. Restricted cubic spline models assessed potential nonlinearity. Results: Among 923 participants, higher serum 25(OH)D concentrations were modestly and linearly associated with lower SBP, DBP, and MAP after multivariable adjustment. Each 10 ng/mL increase in serum 25(OH)D was associated with a 1.07 mmHg lower SBP, a 1.19 mmHg lower DBP, and a 1.22 mmHg lower MAP. Associations remained consistent in sensitivity and subgroup analyses. Conclusions: In young adults, higher serum 25(OH)D concentrations were modestly associated with lower blood pressure indices. These findings confirm prior observational evidence of modest inverse associations between serum 25-hydroxyvitamin D concentrations and blood pressure and extend these observations to a relatively healthy young adult population. Prospective studies are required to clarify temporality and clinical relevance.

## 1. Introduction

Blood pressure is a continuous determinant of cardiovascular risk across the life course [1,2], with elevations in early adulthood tracking into midlife contributing to long-term cardiovascular morbidity [3]. Even in the absence of overt hypertension, modest increases in blood pressure during young adulthood may reflect early vascular dysfunction and represent a critical window for primordial prevention [4,5].

Vitamin D has been proposed as a modulator of blood pressure through several biological pathways, including suppression of the renin–angiotensin–aldosterone system (RAAS) [6,7,8,9], regulation of vascular smooth muscle tone [9,10], enhancement of endothelial function [11], and anti-inflammatory effect [12,13,14]. Numerous observational studies across the life course [15,16,17] have reported inverse associations between serum 25-hydroxyvitamin D [25(OH)D] concentrations and blood pressure or hypertension risk [18,19]. However, effect sizes in these studies have generally been modest, and findings have not been fully consistent across populations. In contrast, randomized trials of vitamin D supplementation have not consistently demonstrated clinically meaningful reduction in blood pressure [20].

Young adulthood represents a distinct cardiometabolic stage characterized by preserved vascular compliance [21,22], and low prevalence of overt comorbidity or antihypertensive treatment. Although vitamin D−blood pressure associations have been examined in adult populations, many prior studies have included broad age ranges or high-risk cohorts. The present study provided age-specific data from a relatively healthy young adult population, enabling evaluation of these associations within an early life-stage context.

In this context, we examined the association between serum 25(OH)D concentrations and systolic blood pressure (SBP), diastolic blood pressure (DBP), and mean arterial pressure (MAP), in a community-based cohort of adults aged 18–45 years. We also assessed whether these associations were linear across the observed range of vitamin D concentrations.

## 2. Methods

### 2.1. Study Design and Population

This study was conducted within the Young Taiwanese Adults (YOTA) cohort, a population-based cohort designed to investigate cardiometabolic risk factors across early life stages. The YOTA cohort consists of two components. The original YOTA cohort was prospectively established between 2006 and 2008 through a nationwide hypertension screening program and enrolled children and adolescents younger than 18 years. The YOTA cohort has been described in detail previously [23].

Between 2017 and 2019, a new adult cohort, referred to as the NEW YOTA cohort, was assembled. This cohort included individuals followed from the original YOTA cohort as well as newly recruited adults identified through a nationwide obesity screening program. The final NEW YOTA sample consisted of 1034 participants: 542 followed from the original cohort and 492 newly enrolled adults.

**Figure 1:**
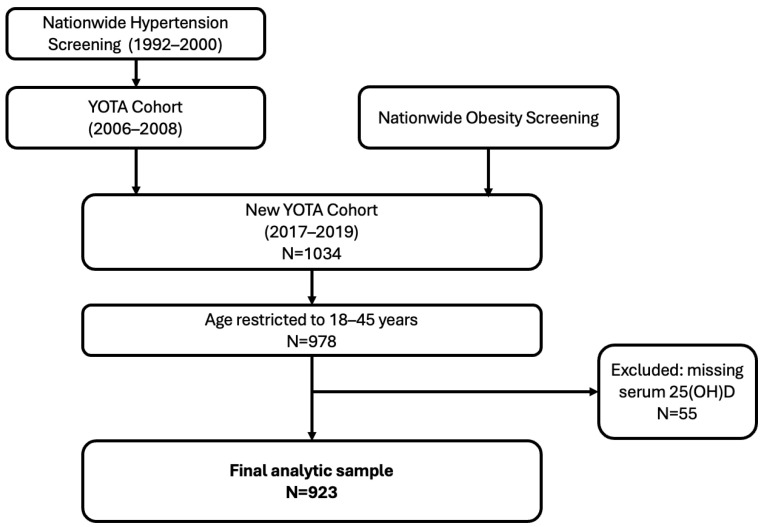
Formation of the Young Taiwanese Adults (YOTA) cohort and participant selection for the present analysis. The Young Taiwanese Adults (YOTA) cohort was established based on nationwide hypertension screening conducted between 1992 and 2000 and a nationwide obesity screening program. The New YOTA cohort was recruited between 2017 and 2019. For the present analysis, participants were restricted to adults aged 18–45 years. Individuals with missing serum 25-hydroxyvitamin D [25(OH)D] measurements were excluded, resulting in a final analytic sample of 923 participants. Abbreviations: YOTA, Young Taiwanese Adults; 25(OH)D, 25-hydroxyvitamin D.

For the present analysis, we conducted cross-sectional analyses using data from the 2017–2019 of the NEW YOTA cohort. To focus on early adulthood and reduce confounding by age-related comorbidities, we restricted the analytic sample to participants aged 18–45 years with available data on serum 25(OH)D and blood pressure measurements. The study was approved by the Institutional Review Board of National Taiwan University Hospital (IRB No. 201604089RINA), and written informed consent was obtained from all participants. Individuals with active cancer, cognitive impairment, or who declined participation were excluded [24]. The formation of the Young Taiwanese Adults (YOTA) cohort and the participant selection process for the present analysis are illustrated in Figure 1. The upper age limit of 45 years was selected to focus on early adulthood while minimizing confounding from age-related vascular stiffening, increasing cardiometabolic comorbidity, and higher prevalence of antihypertensive treatment after midlife.

### 2.2. Assessment of Serum 25-Hydroxyvitamin D

Fasting venous blood samples were collected and stored at −80 °C until analysis. Serum 25-hydroxyvitamin D [25(OH)D] concentrations, defined as the sum of 25(OH)D_2_ and 25(OH)D_3_, were measured using the TOTAL Liaison chemiluminescent immunoassay (Liaison, Diasorin S.p.A., Saluggia, Italy) [25]. The assay demonstrated a coefficient of variation of 2.65% (SD = 1.2%) [26]. Recruitment was limited to March–September to minimize seasonal variation. Serum 25(OH)D was modeled continuously (per 10 ng/mL increase) and categorized using commonly applied epidemiological cut points (<12, 12–20, 20–30, and ≥30 ng/mL) to facilitate comparability with prior literature [27].

### 2.3. Blood Pressure Measurement

Blood pressure was measured using a cuff sphygmomanometer equipped with an oscillometric device (DynaPulse 200M, Pulse Metric Inc., San Diego, CA, USA) [28]. Measurements were obtained from both arms after at least 5 min of seated rest in a quiet environment, and the average of two measurements was used for analyses. The device derives central systolic, diastolic, and mean arterial pressure from brachial arterial waveforms using pulse-waveform analysis, as previously described and validated against invasive and noninvasive measurements [29]. The DynaPulse system has been used in prior studies conducted by our group [30,31]. Hypertension was defined as SBP ≥ 130 mmHg and/or DBP ≥ 80 mmHg, or self-reported physician diagnosis [32].

### 2.4. Covariates

Covariates were selected a priori based on biological plausibility and prior literature. Demographic variables included age and sex. Anthropometric measures included body mass index (BMI), calculated as weight (kg) divided by height squared (m^2^). Cardiometabolic factors assessed at the 2017–2019 examination and included in multivariable models comprised serum creatinine, glycated hemoglobin (HbA1c), low-density lipoprotein cholesterol (LDL-C), total cholesterol, and serum albumin concentration. Hyperlipidemia was defined as low-density lipoprotein cholesterol (LDL-C) ≥ 130 mg/dL, total cholesterol ≥ 200 mg/dL, triglycerides ≥ 200 mg/dL, or high-density lipoprotein cholesterol (HDL-C) <40 mg/dL in men or <50 mg/dL in women. Lifestyle factors included cigarette smoking and regular exercise, as assessed by standardized questionnaires.

### 2.5. Statistical Analysis

Continuous variables are presented as means ± standard deviations, and categorical variables as frequencies and percentages. Between-group comparisons were performed using Student’s *t* test or χ^2^ test, as appropriate. Associations between serum 25(OH)D concentrations and blood pressure indices were evaluated using multivariable linear regression.

Four sequential models were constructed: Model 1, an unadjusted (crude) model; Model 2, adjusted for age and sex; Model 3, further adjusted for body mass index, serum creatinine level, glycated hemoglobin (HbA1c), low-density lipoprotein cholesterol, total cholesterol, and serum albumin concentration; and Model 4, further adjusted for cigarette smoking and regular exercise.

To further account for socioeconomic variation, an additional sensitivity model included educational attainment (university degree or above vs. below university level), as a proxy for socioeconomic status.

Serum 25(OH)D was modeled as a continuous variable and scaled per 10 ng/mL increase. Regression coefficients (β) and 95% confidence intervals (CIs) were reported for each blood pressure outcome.

Sensitivity analyses were conducted using the fully adjusted model after excluding participants with diabetes mellitus, hypertension, or both, to minimize potential confounding from established cardiometabolic disease or treated hypertension.

Potential nonlinearity in the associations between serum 25(OH)D and blood pressure parameters was assessed using restricted cubic spline functions with 3 knots. Two-sided *p* values < 0.05 were considered statistically significant.

All statistical analyses were performed using R software (R Foundation for Statistical Computing, Vienna, Austria; version 4.5.2). The tidyverse package suite was used for data management and descriptive analyses, stats for linear regression modeling, and rms for restricted cubic spline analyses.

## 3. Results

### 3.1. Study Population

After restricting the cohort to adults aged 18–45 years, 978 participants were eligible for analysis. Of these, 55 individuals were excluded due to missing serum 25-hydroxyvitamin D measurements, resulting in a final analytic sample of 923 participants with available vitamin D and blood pressure data (Figure 1).

Baseline characteristics stratified by categories of serum 25(OH)D concentrations are presented in Table 1. Participants with higher 25(OH)D concentrations tended to be older and more likely to be male. Additional biochemical, metabolic, lifestyle, and comorbidity characteristics are provided in Supplement Table S1. Serum 25(OH)D concentrations were categorized as <12, 12–20, 20–30, and ≥30 ng/mL [27].

### 3.2. Association Between Serum 25(OH)D and Blood Pressure

Multivariable linear regression analyses evaluating the associations between serum 25-hydroxyvitamin D concentrations and blood pressure parameters are presented in Table 2. In the unadjusted model (Model 1), serum 25(OH)D concentrations were not significantly associated with systolic blood pressure (SBP), diastolic blood pressure (DBP), or mean arterial pressure (MAP). After adjustment for age and sex (Model 2), higher serum 25(OH)D concentrations were significantly associated with lower SBP, DBP, and MAP. These inverse associations remained statistically significant after further adjustment for body mass index, serum creatinine level, glycated hemoglobin (HbA1c), low-density lipoprotein cholesterol, total cholesterol, and serum albumin concentration (Model 3), and were not materially altered after additional adjustment for cigarette smoking and regular exercise (Model 4).

In Model 4, each 10 ng/mL increase in serum 25(OH)D was associated with a 1.07 mmHg lower SBP (95% CI, −1.96 to −0.19; *p* = 0.017), a 1.19 mmHg lower DBP (95% CI, −1.84 to −0.54; *p* < 0.001), and a 1.22 mmHg lower MAP (95% CI, −1.92 to −0.53; *p* < 0.001).

Although the magnitude of associations attenuated modestly with sequential covariate adjustment, the inverse relationships remained statistically significant in the fully adjusted model, suggesting that the observed associations were robust to adjustment for key confounders.

Restricted cubic spline analyses supported a linear relationship between serum 25(OH)D concentrations and blood pressure indices. Although the overall associations with DBP (*p* = 0.004) and MAP (*p* = 0.005) were statistically significant, the *p* values for nonlinearity were >0.05 for all outcomes (SBP, DBP, and MAP), indicating no evidence of departure from linearity (Table 3). These findings suggest that the associations between 25(OH)D and blood pressure parameters are approximately linear across the observed range.

### 3.3. Sensitivity Analysis

In sensitivity analyses excluding participants with diabetes mellitus (DM), hypertension (HTN), or both, the inverse associations between serum 25(OH)D concentrations and SBP, DBP, and MAP remained statistically significant and of similar magnitude. These results suggest that the observed relationships were unlikely to be fully explained by participants meeting hypertension criteria (Table 4). In the fully adjusted model, each 10 ng/mL increase in serum 25(OH)D was associated with a 0.90 to 1.00 mmHg lower SBP, a 1.15 to 1.19 mmHg lower DBP, and a 1.12 to 1.22 mmHg lower MAP, depending on the exclusion criteria. These findings suggest that the observed associations were not driven by participants with established cardiometabolic conditions.

Additional adjustment for education level likewise did not materially alter the associations between serum 25(OH)D and blood pressure parameters, suggesting that measured socioeconomic variation did not substantially confound the observed associations (Supplementary Table S2).

## 4. Discussion

In this cohort of adults aged 18–45 years, higher serum 25(OH)D concentrations were inversely associated with systolic, diastolic and mean arterial blood pressure. These associations remained statistically significant after comprehensive multivariable adjustment and sensitivity analyses excluding participants with diabetes mellitus, hypertension, or both. Although the magnitude of associations was modest, the consistency across pressure indices and sensitivity analyses supports the presence of a modest inverse association between vitamin D status and blood pressure in young adulthood. Overall, our findings should be interpreted as confirmatory evidence within a younger population rather than as a novel demonstration of association.

### 4.1. Clinical Implications and Guideline Context

From a clinical perspective, the observed effect sizes do not support vitamin D supplementation as a primary antihypertensive therapy. This interpretation is consistent with randomized controlled trials [33], and with current clinical guidelines, including the 2024 Endocrine Society Clinical Practice Guideline on vitamin D supplementation [34]. The guideline emphasizes that vitamin D supplementation should be targeted toward individuals with deficiency for skeletal and selected extra-skeletal outcomes and does not recommend supplementation solely for blood pressure reduction or cardiovascular disease prevention in the general population. Nevertheless, the guideline also acknowledges that vitamin D deficiency is common and may be associated with adverse cardiometabolic phenotypes, particularly in younger populations with prolonged exposure [34]. Recent expert consensus published in Nutrients further emphasizes individualized assessment of vitamin D status within cardiovascular prevention frameworks, while cautioning against overinterpretation of observational findings [35].

Within this framework, our findings support the interpretation of serum 25(OH)D as a biomarker associated with early vascular phenotype rather than as evidence supporting therapeutic intervention for blood pressure reduction. Although the absolute magnitude of association is modest at the individual level, even small shifts in blood pressure distribution during early adulthood may have potential relevance within primordial prevention frameworks.

### 4.2. Physiological Interpretation and Potential Mechanisms

In young adults, arterial stiffening is minimal, and blood pressure regulation is more strongly influenced by peripheral vascular resistance and microvascular function. The observed associations with diastolic blood pressure and mean arterial pressure may therefore be compatible with differences in microvascular hemodynamic profiles rather than large-artery structural changes [5].

Experimental studies have described biological pathways that may plausibly relate vitamin D signaling to vascular function, including modulation of the renin–angiotensin–aldosterone system [6]. Activation of the vitamin D receptor in endothelial and vascular smooth muscle cells has been associated with enhanced nitric oxide bioavailability, reduced oxidative stress, and modulation of inflammatory signaling [12]. Prior studies have also reported anti-inflammatory and cardiometabolic correlates of vitamin D status [36,37]. These observations align with prior reports describing associations between microvascular function and blood pressure phenotypes [38].

Although conventional brachial blood pressure measurements primarily reflect peripheral systolic and diastolic values, central blood pressure incorporates arterial waveform characteristics and may more closely reflect hemodynamic load on central organs [39]. In the present study, inverse associations were observed across systolic, diastolic, and mean arterial pressure indices. These findings are compatible with prior evidence linking vascular function and microvascular hemodynamics to blood pressure phenotypes. However, whether the biological pathways described in experimental studies explain the associations observed in this population-based analysis remains uncertain. Given the cross-sectional design, mechanistic inference cannot be established, and the observed associations should not be interpreted as evidence of a direct physiological effect of vitamin D on blood pressure regulation.

### 4.3. Comparison with Prior Studies and Life-Course Perspective

Our findings are consistent with previous observational studies reporting inverse associations between vitamin D and blood pressure [40], although most prior studies were conducted in older or higher-risk populations [18,19]. In contrast, randomized trials of vitamin D supplementation have generally failed to demonstrate clinically meaningful blood pressure reductions, contributing to ongoing debate regarding causality [41,42]. Differences in population characteristics and vascular phenotype may contribute to discrepancies between observational and randomized trial findings. In young adults with preserved vascular function, vitamin D–blood pressure associations may be modest and more evident for DBP and MAP, reflecting microvascular regulation rather than large artery pathology.

Notably, the linear associations observed in our study contrast with nonlinear or threshold relationships reported in older cohorts, suggesting that the association may differ across stages of the life course [20,40,43]. A prior cohort study from northern Taiwan reported higher mean 25(OH)D concentrations and a lower prevalence of deficiency than observed in our cohort, yet demonstrated similar age- and sex-related patterns [44]. Together, these findings are consistent with prior observational evidence. The principal contribution of the present study is confirmatory: it indicates that the modest inverse associations between serum 25(OH)D and blood pressure previously reported in older or higher-risk populations are also observable in a younger, relatively healthy community-based cohort.

### 4.4. Robustness of Findings

The robustness of our findings was supported by multiple sensitivity analyses. Inverse associations between serum 25(OH)D and blood pressure parameters remained consistent after exclusion of participants with diabetes mellitus, hypertension, or both, indicating that the results were not driven by individuals with established cardiometabolic disease. Effect estimates were stable across alternative model specifications, and no statistically significant interactions were observed in subgroup analyses stratified by body mass index [45,46] or sex [47] suggesting no statistically significant effect modification within this age range (Table 5).

### 4.5. Strengths and Limitations

This study has several strengths, including the use of a well-characterized, community-based cohort with standardized assessments of serum 25(OH)D and blood pressure, evaluation of multiple blood pressure indices, and comprehensive covariate adjustment. Restricting the analytic sample to adults aged 18–45 years allowed focused investigation of early blood pressure regulation while minimizing confounding by age-related comorbidities and antihypertensive medication use [48].

Several limitations should be acknowledged. Although key behavioral factors including smoking status and exercise were adjusted for, and educational attainment was considered as a proxy for socioeconomic status, residual confounding from unmeasured determinants cannot be entirely excluded. Dietary sodium intake was not assessed with high precision. Although habitual dietary patterns were collected using a food frequency questionnaire, discretionary salt use and sodium from processed or externally prepared foods were not specifically quantified. As these sources may constitute a substantial proportion of total sodium exposure, omission of such components could have resulted in systematic underestimation and non-differential exposure misclassification. Therefore, measurement error in sodium assessment and incomplete adjustment for sodium intake cannot be entirely excluded.

Recruitment was restricted to March through September to reduce seasonal variation in serum 25(OH)D concentrations; however, individual-level sunlight exposure behaviors were not directly measured. Detailed antihypertensive medication data were not available; however, exclusion of participants with hypertension yielded similar results, suggesting that treatment-related blood pressure lowering is unlikely to fully explain the observed associations.

Finally, although the observed associations were statistically significant, their magnitude was modest and may not translate into immediate clinical implications at the individual level. Given the cross-sectional design, causality cannot be inferred, and serum 25(OH)D should be interpreted as a biomarker associated with vascular phenotype rather than a determinant of blood pressure regulation. Longitudinal studies are needed to determine whether vitamin D–related differences in blood pressure during early adulthood track into later cardiovascular risk.

## 5. Conclusions

In adults aged 18–45 years, higher serum 25-hydroxyvitamin D concentrations were modestly and linearly associated with lower systolic, diastolic, and mean arterial blood pressure. These findings confirm and contextualize prior observational evidence within a young adult population. Whether these associations translate into long-term cardiovascular benefit requires longitudinal investigation.

## Figures and Tables

**Table 1:** Baseline characteristics of participants according to serum 25-hydroxyvitamin D categories.

		25(OH)D Groups, ng/mL				
Variables	Overall N = 923	<12 N = 143 (13.9%)	12–20 N = 346 (37.5%)	20–30 N = 352 (36.8%)	≥30 N = 82 (9.2%)	*p* for Trend
Serum 25(OH)D, ng/mL	19.9 ± 8.1	8.7 ± 2.7	16.3 ± 2.3	24.3 ± 2.7	36.0 ± 7.3	<0.001
Age	31.9 ± 4.5	31.1 ± 4.5	31.6 ± 4.4	32.3 ± 4.6	32.7 ± 4.3	0.010
Male, *n* (%)	408 (44.2)	46 (32.2)	115 (33.2)	171 (48.6)	52 (63.4)	<0.001
BSA, m^2^	1.7 ± 0.2	1.7 ± 0.2	1.7 ± 0.2	1.7 ± 0.2	1.8 ± 0.2	0.002
BMI, kg/m^2^	23.0 ± 4.3	23.3 ± 4.9	22.9 ± 4.3	23.0 ± 4.01	23.6 ± 4.7	0.531
Creatinine, mg/dL	0.9 ± 0.9	0.9 ± 1.6	0.9 ± 1.1	0.8 ± 0.2	0.9 ± 0.2	0.715
ALT, U/dL	19.5 ± 13.0	21.4 ± 15.4	18.6 ± 10.7	19.5 ± 12.2	21.9 ± 21.3	0.075
Hb, g/dL	14.1 ± 1.8	13.8 ± 2.0	13.9 ± 1.7	14.4 ± 1.9	14.5 ± 1.6	<0.001
AC sugar, mg/dL	86.8 ± 20.1	86.0 ± 21.5	88.9 ± 28.8	85.4 ± 9.2	86.3 ± 10.0	0.151
hsCRP, mg/dL	0.2 ± 0.8	0.1 ± 0.2	0.2 ± 0.3	0.2 ± 0.7	0.4 ± 2.1	0.017
Smoker (%)	132 (14.3)	11 (7.7)	46 (13.3)	50 (14.2)	18 (22.2)	0.023
Alcohol drinker (%)	305 (33.0)	38 (26.8)	101(29.3)	120 (35.0)	29 (37.7)	0.138
Regular supplement (%)	117 (12.7)	12 (8.4)	37 (10.7)	47 (13.4)	11 (13.4)	0.387
SBP, mmHg	115.5 ± 13.9	116.1 ± 15.8	115.5 ± 13.0	115.0 ± 13.7	117.0 ± 14.9	0.656
DBP, mmHg	65.7 ± 9.6	66.5 ± 11.4	65.8 ± 8.7	65.3 ± 9.4	65.9 ± 10.1	0.617
MAP, mmHg	81.1 ± 10.6	81.9 ± 12.5	81.3 ± 9.8	80.5 ± 10.4	81.3 ± 11.4	0.585
Pulse Pressure, mmHg	49.8 ± 7.8	49.6 ± 8.1	49.7 ± 7.7	49.7 ± 7.8	51.0 ± 8.3	0.522
Heart Rate, bpm	70.4 ± 9.6	71.0 ± 9.54	71.5 ± 9.7	69.6 ± 9.2	68.6 ± 10.4	0.013

Data are presented as mean ± standard deviation for continuous variables and number (percentage) for categorical variables. Serum 25-hydroxyvitamin D concentrations were categorized as <12, 12–20, 20–30, and ≥30 ng/mL. *p* for trend was calculated using linear regression for continuous variables and the Cochran–Armitage trend test for categorical variables. Abbreviations: 25(OH)D, 25-hydroxyvitamin D; BSA, body surface area; BMI, body mass index; ALT, alanine aminotransferase; Hb, hemoglobin; AC sugar, fasting glucose; hsCRP, high-sensitivity C-reactive protein; SBP, systolic blood pressure; DBP, diastolic blood pressure; MAP, mean arterial pressure.

**Table 2:** Multivariable linear regression analyses of serum 25-hydroxyvitamin D and blood pressure parameters.

	SBP		DBP		MAP	
	β per 10 ng/mL (95% CI)	*p* Value	β per 10 ng/mL (95% CI)	*p* Value	β per 10 ng/mL (95% CI)	*p* Value
Model 1	0.04 (−1.07, 1.15)	0.947	−0.56 (−1.32, 0.20)	0.151	−0.56 (−1.41, 0.28)	0.191
Model 2	−1.74 (−2.76, −0.73)	<0.001	−1.61 (−2.34, −0.89)	<0.001	−1.75 (−2.56, −0.95)	<0.001
Model 3	−1.20 (−2.09, −0.31)	0.008	−1.26 (−1.92, −0.61)	<0.001	−1.31 (−2.00, −0.61)	<0.001
Model 4	−1.07 (−1.96, −0.19)	0.017	−1.19 (−1.84, −0.54)	<0.001	−1.22 (−1.92, −0.53)	<0.001

Data are presented as β coefficients (95% confidence intervals) per 10 ng/mL increase in serum 25-hydroxyvitamin D concentration. SBP, systolic blood pressure; DBP, diastolic blood pressure; MAP, mean arterial pressure. Model 1: Unadjusted (crude) model. Model 2: Adjusted for age and sex. Model 3: Further adjusted for body mass index, serum creatinine level, glycated hemoglobin (HbA1C), low-density lipoprotein cholesterol, total cholesterol, and serum albumin concentration. Model 4: Further adjusted for cigarette smoking and regular exercise.

**Table 3:** Assessment of non-linear associations between serum 25-hydroxyvitamin D and blood pressure parameters.

Outcome	*p* for Overall	*p* for Non-Linear
SBP	0.094	0.705
DBP	0.004	0.800
MAP	0.005	0.683

Nonlinearity was assessed using restricted cubic spline models adjusted for age, sex, body mass index, serum creatinine level, glycated hemoglobin (HbA1c), low-density lipoprotein cholesterol, total cholesterol, serum albumin concentration, cigarette smoking, and regular exercise. *p* values for overall associations and for nonlinearity were derived from likelihood ratio tests comparing models with and without spline terms. Abbreviations: SBP, systolic blood pressure; DBP, diastolic blood pressure; MAP, mean arterial pressure.

**Table 4:** Sensitivity analyses of the associations between serum 25-hydroxyvitamin D and blood pressure parameters after exclusion of participants with diabetes mellitus and/or hypertension.

Outcome	Model	Main (All) N = 923	Exclude DM N = 906	Exclude HTN N = 896	Exclude DM + HTN N = 885
SBP	Model 4	−1.07 (−1.96, −0.19)	−1.00 (−1.88, −0.12)	−0.96 (−1.80, −0.13)	−0.90 (−1.73, −0.06)
DBP	Model 4	−1.19 (−1.84, −0.54)	−1.19 (−1.84, −0.54)	−1.19 (−1.79, −0.58)	−1.15 (−1.76, −0.54)
MAP	Model 4	−1.22 (−1.92, −0.53)	−1.19 (−1.88, −0.50)	−1.17 (−1.82, −0.52)	−1.12 (−1.77, −0.47)

Data are presented as β coefficients (95% confidence intervals) per 10 ng/mL increase in serum 25-hydroxyvitamin D. Sensitivity analyses were conducted using the fully adjusted model (Model 4) after exclusion of participants with diabetes mellitus (DM), hypertension (HTN), or both. Model 4 was adjusted for age, sex, body mass index, serum creatinine level, glycated hemoglobin (HbA1c), low-density lipoprotein cholesterol, total cholesterol, serum albumin concentration, cigarette smoking, and regular exercise. Abbreviations: DM, diabetes mellitus; HTN, hypertension; SBP, systolic blood pressure; DBP, diastolic blood pressure; MAP, mean arterial pressure.

**Table 5:** Subgroup analyses by body mass index and sex.

Outcome	BMI Subgroup			Sex Subgroup		
	β (95% CI), BMI < 24	β (95% CI), BMI ≥ 24	*p* for Interaction	β (95% CI), Female	β (95% CI), Male	*p* for Interaction
SBP	−0.95 (−1.96, 0.05)	−1.45 (−3.22, 0.33)	0.912	−1.52 (−2.70, −0.34)	−0.56 (−1.89, 0.77)	0.531
DBP	−1.07 (−1.75, −0.39)	−1.69 (−3.09, −0.30)	0.758	−1.03 (−1.86, −0.20)	−1.30 (−2.34, −0.26)	0.654
MAP	−1.11 (−1.84, −0.37)	−1.64 (−3.12, −0.17)	0.879	−1.21 (−2.11, −0.31)	−1.17 (−2.25, −0.08)	0.884

Data are presented as β coefficients (95% confidence intervals) per 10 ng/mL increase in serum 25-hydroxyvitamin D. Subgroup analyses were conducted stratified by body mass index (BMI < 24 vs. ≥24 kg/m^2^) and sex. All models were adjusted for age, sex (except in sex-stratified analyses), body mass index (except in BMI-stratified analyses), serum creatinine level, glycated hemoglobin (HbA1c), low-density lipoprotein cholesterol, total cholesterol, serum albumin concentration, cigarette smoking, and regular exercise. *p* values for interaction were derived from multiplicative interaction terms between serum 25-hydroxyvitamin D and the corresponding subgroup variable. Abbreviations: SBP, systolic blood pressure; DBP, diastolic blood pressure; MAP, mean arterial pressure; BMI, body mass index.

## Data Availability

The data presented in this study are available on reasonable request from the corresponding author. The data are not publicly available due to ethical restrictions and the protection of participant privacy in accordance with institutional review board requirements.

## Supplementary Materials

The following supporting information can be downloaded at: https://www.mdpi.com/article/10.3390/nu18050876/s1. Table S1. Extended baseline characteristics by vitamin D categories. Table S2. Multivariable linear regression analyses additionally adjusted for education level.
