# Supplementary material for: Association Between Serum 25-Hydroxyvitamin D and Blood Pressure in Young Adults
Source: Nutrients. 2026 Mar 9;18(5):876. doi: 10.3390/nu18050876 (PMC12987020; doi:10.3390/nu18050876)
Supplement: Supplementary file 1 [file nutrients-18-00876-s001.zip › nutrients-4117479-supplementary.pdf]

Supplementary Materials

Supplementary Table S1. Extended baseline characteristics by vitamin D categories.

Variables	Overall N= 978	25(OH)D groups, ng/mL <12 N=143 (15.4%)	12-20 N=346 (37.4%)	20-30 N= 352 (38.3%)	≥ 30 N= 82 (8.9%)	*p* for trend
**A. Biochemical and metabolic parameters**						
Waist circumference, cm	79.8 ± 13.4	80.2 ± 13.8	79.3 ± 12.9	79.8 ± 13.7	82.04 ± 14.17	0.402
Insulin, µU/mL	10.6 ± 6.1	11.4 ± 8.0	10.4 ± 5.7	10.1 ± 4.9	11.9 ± 8.4	0.033
HOMA-IR	2.3 ± 1.8	2.5 ± 2.2	2.4 ± 2.2	2.2 ± 1.2	2.6 ± 2.2	0.103
Alb, g/dL	4.7 ± 0.3	4.7 ± 0.3	4.7 ± 0.3	4.7 ± 0.2	4.7 ± 0.3	0.279
TP, g/dL	7.5 ± 0.5	7.6 ± 0.7	7.5 ± 0.4	7.5 ± 0.4	7.6 ± 0.4	0.317
TCHO, mg/dl	188.2 ± 41.6	191.9 ± 47.3	192.3 ± 48.5	185.4 ± 33.9	175.9 ± 30.8	0.004
TG, mg/dl	100.1 ± 255.2	155.3 ± 647.9	89.8 ± 67.5	92.1 ± 63.9	84.6 ± 61.3	0.058
HDL, mg/dl	59.5 ± 15.1	61.3 ± 17.8	61.2 ± 15.5	57.8 ± 13.7	57.1 ± 14.0	0.004
LDL, mg/dl	114.8 ± 36.5	112.9 ± 34.4	118.3 ± 45.0	114.1 ± 29.9	105.0 ± 26.7	0.024
**B. Renal and mineral metabolism**						
BUN, mg/dL	13.5 ± 4.6	13.1 ± 5.7	13.4 ± 5.5	13.3 ± 3.1	14.1 ± 3.5	0.433
Uric acid, mg/dL	5.6 ± 1.5	5.6 ± 1.6	5.5 ± 1.5	5.8 ± 1.5	5.9 ± 1.3	0.008
CysC, mg/L	0.9 ± 3.5	0.8 ± 0.64	1.0 ± 3.6	1.0 ± 4.7	0.8 ± 0.1	0.921
Ca, mg/dL	9.4 ± 1.3	9.4 ± 0.6	9.3 ± 0.4	9.4 ± 2.2	9.4 ± 0.4	0.496
P, mg/dL	3.7 ± 3.1	3.6 ± 0.8	3.6 ± 0.6	3.8 ± 5.1	3.5 ± 0.6	0.920
**C. Life style and social economic factors**						
Exercise habits (%)	565 (57.8)	65 (45.5)	198 (57.4)	221 (62.8)	49 (59.8)	0.005
University (%)	804 (82.2)	120 (83.9)	294 (85.0)	281 (79.8)	69 (84.1)	0.314
BMR	1439.9 ± 256.5	1415.3 ± 262.8	1406.2 ± 247.6	1460.5 ± 257.4	1534.5 ± 259.4	<0.001
TEE, kcal/day	2262.2 ± 534.5	2170.8 ± 498.9	2192.5 ± 512.7	2307.1 ± 530.8	2514.2 ± 614.6	<0.001
**D. Comorbidities**						
DM (%)	15 (1.5)	2 (1.4)	9 (2.6)	2 (0.6)	2 (2.4)	0.179
Hypertension (%)	26 (2.7)	7 (4.9)	8 (2.3)	10 (2.8)	1 (1.2)	0.342
Hyperlipidemia (%)	12 (1.2)	1 (0.7)	7 (2.0)	3 ± 0.9	1 (1.2)	0.497

Data are presented as mean ± standard deviation for continuous variables and number (percentage) for categorical variables. Serum 25-hydroxyvitamin D categories were defined as <12, 12–20, 20–30, and ≥30 ng/mL. *P* values for trend

were calculated using linear regression for continuous variables and the Cochran–Armitage test for trend for categorical variables.

Abbreviations: HOMA-IR, homeostasis model assessment of insulin resistance; HDL, high-density lipoprotein cholesterol; LDL, low-density lipoprotein cholesterol; TCHO, total cholesterol; TG, triglycerides; BUN, blood urea nitrogen; CysC, cystatin C; Ca, calcium; P, phosphorus; BMR, basal metabolic rate; TEE, total energy expenditure; DM, diabetes mellitus.

Supplementary Table S2. Multivariable linear regression analyses additionally adjusted for education level

	Model 4 (Main Analysis)		Model 5 (Model 4+ University Education)	
Outcome	β per 10 ng/mL (95% CI)	*p* value	β per 10 ng/mL (95% CI)	*p* value
SBP	−1.07(−1.95, −0.19)	0.017	−1.02(−1.91, −0.14)	0.024
DBP	−1.19(−1.84, −0.54)	<0.001	−1.16(−1.81, −0.51)	<0.001
MAP	−1.22(−1.92, −0.53)	<0.001	−1.17(−1.86, −0.47)	0.001

Data are presented as β coefficients (95% confidence intervals) per 10 ng/mL increase in serum 25-hydroxyvitamin D concentration.

Model 4 was adjusted for age, sex, body mass index, serum creatinine level, glycated hemoglobin (HbA1c), low-density lipoprotein cholesterol, total cholesterol, serum albumin concentration, cigarette smoking, and regular exercise.

Model 5 was additionally adjusted for education level (binary variable: university degree or above vs. below university level), as a proxy for socioeconomic status.
